# *Bacillus subtilis* extracellular vesicles surface-displaying superoxide dismutase attenuate alcoholic liver injury by activating the Nrf2/HO-1 axis

**DOI:** 10.1186/s40643-026-01112-6

**Published:** 2026-09-12

**Authors:** Baoxian Li, Jiali Chen, Chaozhi Wei, You Wei, Chunqiang Pan, Tao Liu, Qingchi Wang, Shiyu Li, Yao Zhao, Mengyu Zhang, Chenfan Sun, Chengran Guan, Domenico Nuzzo, Jintao Cheng, Yuanxiang Jin, Guiling Yang

**Affiliations:** 1 College of Biotechnology and Bioengineering, Zhejiang University of Technology, Hangzhou, 310032 China; 2 Xianghu Laboratory, Hangzhou, 311231 China; 3 Institute of Agro-Product Safety and Nutrition, Zhejiang Academy of Agricultural Sciences, Hangzhou, 310021 China; 4 Zhejiang Key Laboratory of Public Health Detection and Pathogenesis Research, Department of Microbiology, Zhejiang Provincial Center for Disease Control and Prevention, Hangzhou, 310051 China; 5 Key Lab of Dairy Biotechnology and Safety Control, College of Food Science and Engineering, Yangzhou University, Jiangsu, 225127 China; 6 Istituto per la Ricerca e l’Innovazione Biomedica, CNR, via U. La Malfa 153, 90146 Palermo, Italy; 7 Present Address: Xianghu Laboratory, Chuangye Valley Building, No. 168, Gengwen Road, Xiaoshan District, Hangzhou, 311231 China

**Keywords:** Alcoholic liver disease, Bacterial extracellular vesicles, Surface display, Superoxide dismutase, Nrf2/HO-1

## Abstract

Alcoholic liver disease (ALD) represents a globally prevalent progressive hepatic disorder with continuously rising incidence. Its pathological mechanisms mainly stem from the direct toxic effects of alcohol metabolites, accompanied by aggravated oxidative stress, lipid overaccumulation and inflammatory infiltration.. While current therapies that act on these pathogenic mechanisms alleviate symptoms, they are often limited by gastrointestinal adverse effects and long-term hepatic metabolic burden. Bacterial extracellular vesicles (BEVs), emerging as natural carriers of bioactive molecules and mediators of intercellular communication, offer a novel hepatoprotective strategy against alcoholic liver injury. Superoxide dismutase (SOD), as a crucial antioxidant, has been extensively verified for its capacity to eliminate free radicals. Here, we leverage the advantages of BEVs and the multiple effects of SOD, utilizing surface display technology to locate SOD in *Bacillus subtilis* 168-derived EVs (termed SEVs). In vitro simulated digestive fluids experiment confirms the stability and digestive resistance of SEVs, while in vivo biodistribution assays demonstrate the liver-targeting capability. In ethanol-exposed hepatocytes, SEVs significantly attenuate reactive oxygen species (ROS) overproduction and lipid deposition. In a murine ALD model, SEVs administration reduces hepatic steatosis, serum transaminase levels, and inflammatory infiltration. Mechanistically, SEVs activate the Nrf2/HO-1 antioxidant pathway, a key regulator of cellular redox balance and inflammation, thereby counteracting oxidative damage and inflammatory reaction caused by alcohol stimulation. Notably, SEVs exhibit superior biocompatibility without inducing secondary hepatic burden. Our findings emphasize the dual advantages of SEVs as liver-targeted delivery vehicles and multifunctional liver-protective agents, highlighting their translational potential for ALD management.

**Graphical abstract:**

**Figure:**
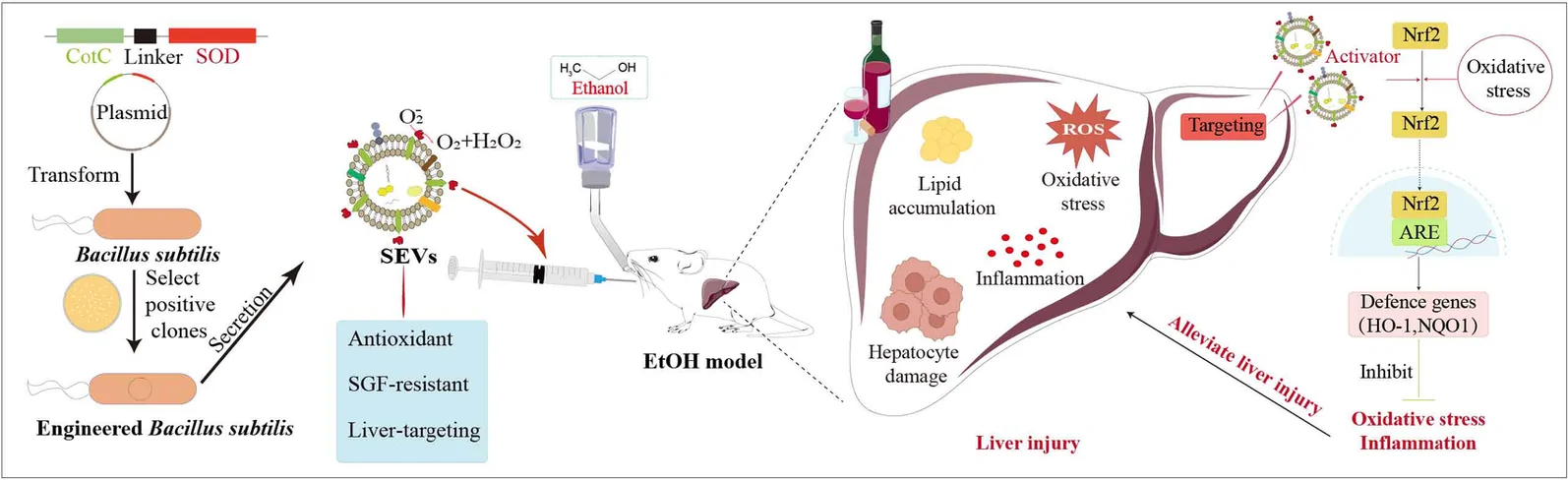

**Supplementary Information:**

The online version contains supplementary material available at https://doi.org/10.1186/s40643-026-01112-6.

## Introduction

ALD, a chronic liver disease caused by prolonged and excessive alcohol consumption, exhibits distinct stepwise progression in clinical manifestations (Mackowiak et al. 2024). In the initial clinical stage, hepatic steatosis and liver injury occur insidiously. Subsequently, it gradually progresses to alcoholic hepatitis, liver fibrosis, and cirrhosis. In severe cases, it may even develop into liver cancer. This disease has become a crucial factor contributing to the death from liver diseases and liver-related diseases worldwide. Since 2014, the prevalence of ALD has been continuously increasing. Particularly during the period of coronavirus disease 2019 (COVID-19) infection, its incidence has significantly increased, thereby placing a substantial burden on the global healthcare system (Åberg et al. 2024).

The liver is the primary organ responsible for alcohol metabolism. Ethanol dehydrogenase and acetaldehyde dehydrogenase work in concert to gradually oxidize alcohol into metabolites such as acetaldehyde and acetic acid. However, long-term excessive alcohol intake overloads the liver's metabolic capacity, leading to a substantial accumulation of toxic metabolites like acetaldehyde. This, in turn, disrupts the redox balance in the liver, triggers inflammatory responses, disturbs the homeostasis of lipid metabolism, and ultimately causes irreversible liver damage (Gao and Bataller. 2011). Currently, the main drugs used for the clinical treatment of ALD, such as silymarin-based drugs, polyene phosphatidylcholine, and reduced glutathione, can alleviate liver oxidative stress and regulate inflammatory responses to a certain extent. Nevertheless, adverse events such as gastrointestinal discomfort and allergic reactions limit the effectiveness of their clinical application (Aghemo et al. 2022; Lai et al. 2024; Mak & Shekhar. 2023; Rambaldi et al. 2005; Shen et al. 2022).

In recent years, bacterial extracellular vesicles (BEVs) have emerged in the fields of health regulation and disease treatment, owing to their unique nanoscale lipid bilayer membrane structure and diverse biomolecular composition (Toyofuku et al. 2018; Xie et al. 2022). These natural vesicles can not only serve as vaccine carriers to stimulate immune responses and enable targeted drug delivery but also possess potential applications as biosensors in the field of biotechnology (Cho et al. 2025; Ji et al. 2024; Kuerban et al. 2020; Liu et al. 2024; Micoli & MacLennan. 2020; Qin et al. 2024; Xu et al. 2022).

This study focused on the surface modification of BEVs, aiming to explore their preventive protective effects on ALD. Based on previous research results (Chen et al. 2025, 2026), we selected the spore coat protein CotC of *Bacillus subtilis* 168 as the anchor protein for the surface display of SOD, which is a crucial antioxidant enzyme and core component of cellular antioxidant defense that scavenges primary ROS to mitigate oxidative stress (Jomova et al. 2024). We achieved the efficient preparation of CotC-SOD EVs (SEVs) by applying tangential flow filtration (TFF) system. SEVs, with their unique lipid bilayer membrane structure and diverse biomolecular composition, were designed to be stable and resistant to digestion in the gastrointestinal tract. In vitro simulated gastrointestinal fluid digestion experiments confirmed this property. Imaging investigation showed that 12 h after gavage, SEVs could successfully reach the liver and remain there for an extended period. Further studies demonstrated that SEVs could effectively alleviate alcohol-induced oxidative stress, lipid deposition, and inflammatory responses, significantly improving the liver injury status in ALD mice via the activation of Nrf2/HO-1 axis.

**Scheme 1:**
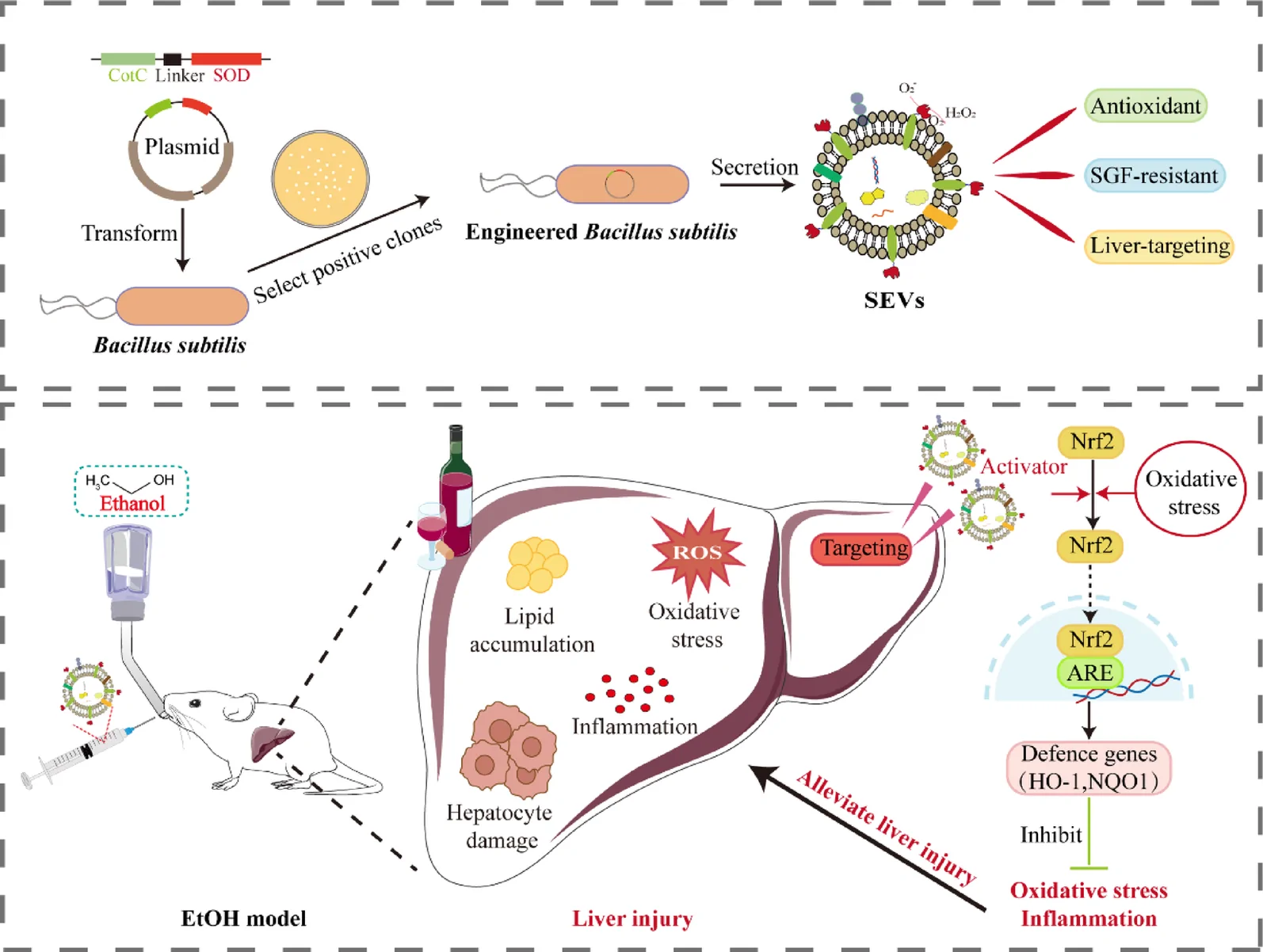
Schematic illustration of Bacillus subtilis 168 engineering and the therapeutic mechanism of SEVs against alcoholic liver injury. Recombinant plasmid pMA09-CotC-SOD-His was transformed into *Bacillus subtilis* 168 to construct a genetically engineered strain, and SEVs were subsequently isolated. SEVs alleviate alcoholic liver injury by activating the Nrf2/HO-1 axis to regulate lipid metabolism, oxidative stress and inflammation

To address the need for safe, oral, live-targeted ALD treatments, and the limitations that current drugs have, such as poor targeting, insufficient antioxidant pathway regulation and side effects, which fail to effectively reduce oxidative stress or restore disrupted antioxidant signaling. We have developed a BEVs-based delivery system (SEVs) displaying SOD with gastrointestinal stability, liver targeting and prolonged retention, and systematically tested its ability to ease ALD-related liver damage via regulating the Nrf2/HO-1 axis. This *Bacillus subtilis*-derived EVs engineering strategy offers new ideas for intervening in refractory diseases. (Scheme 1).

## Materials and methods

### Plasmid construction and bacterial culture

In this study, the recombinant plasmid was constructed via In-Fusion cloning using the CloneExpress One Step Cloning Kit (Vazyme, Nanjing, China). Briefly, the CotC gene and SOD gene were first ligated with a glycine-serine (GS) linker to generate the CotC-GS-SOD fusion fragment, which was then cloned into the pMA09 plasmid. A His-tag was subsequently inserted into the previously constructed vector by PCR amplification, and the final recombinant plasmid (designated pMA09-CotC-SOD-His) was transformed into *E. coli* DH5α competent cells (TSINGKE, Beijing, China) for amplification. Positive clones were validated by colony PCR (primer sequences in Supplementary Table S2) and Sanger sequencing. The verified plasmid was extracted using the FastPure Plasmid Mini Kit (Vazyme, Nanjing, China) following the manufacturer’s protocol, and then transformed into *Bacillus subtilis* 168 (preserved in our laboratory; detailed characteristics in Supplementary Table S1). Target single colonies were screened on Luria–Bertani (LB) broth supplemented with 1‰ kanamycin monosulfate (final concentration 50 μg/mL). The validated positive colony was inoculated into 300 mL of the aforementioned kanamycin-supplemented LB broth and cultured at 37 °C with shaking (200 × g) for 30 h to obtain the recombinant strain (Bahrulolum & Ahmadian. 2025; Kim et al. 2008).

### Isolation and characterization of EVs

Following the methodology described in our prior studies (Chen et al. 2025), the engineered *Bacillus subtilis* 168 fermentation broth was centrifuged at 7,000 × g for 20 min at 4 °C to remove bacterial pellets. The supernatant was further clarified by secondary centrifugation at 12,000 × g for 20 min at 4 °C. The resulting 300 mL supernatant was passed through a 0.45 μm PES filter.

Tangential flow filtration (TFF) was performed using XHEV 1.0, a self-assembled tangential flow filtration system developed in our laboratory. This system was equipped with a 300-kDa MWCO hollow fiber filter composed of a modified PES membrane, operated at a flow rate of 30–50 mL/min, with the transmembrane pressure controlled at approximately 3 psi. The EV-containing sample was circulated in the system, allowing small molecules to pass through the membrane pores and exit via the filtrate outlet. The retentate was diafiltrated with ten volumes of 1 × PBS, and then concentrated to a final volume of 10–15 mL (Jakl et al. 2023; Visan et al. 2022). Finally, the TFF-isolated EVs were subjected to an additional centrifugation at 12,000 × g for 10 min at 4 °C to remove residual debris, followed by sterile filtration through a 0.22-μm PES syringe filter. The purified EVs were stored at − 80 °C until further use.

### Ex vivo digestion of SEVs

To evaluate the gastrointestinal stability of EVs, we referred to a published work and established an in vitro digestion model to simulate the physiological digestion process (Tong et al. 2023). EVs were first incubated with simulated gastric fluid (0.9% w/v sterile physiological saline, pH 3.0, 3 g/L pepsin [1:10000]) at 37 °C for 2 h on a shaker (100 × g). The digestion mixture was then neutralized and supplemented with simulated intestinal fluid components (0.9% w/v sterile physiological saline, pH 8.0, 1 g/L trypsin [1:250], 0.3% w/v bile salts) for intestinal phase simulation (37 °C, 1 h). Digestive juices were eliminated via 100 kDa MWCO ultrafiltration, and particle concentrations were quantified by nanoparticle tracking analysis (NTA) for pre- and post-digestion comparisons.

### Cell culture and chemical treatment

Aml-12 cells were cultured in DMEM with 10% FBS and 1% penicillin–streptomycin (100 μg/mL) at 37 °C and 5% CO₂. 95% ethanol (Macklin, Shanghai, China) and EVs were filter-sterilized (0.22 μm) before use. Cells were seeded at 8,000 cells/well in 96-well plates, incubated for 12 h, and treated with SEVs in serum-free medium for 24 h. Cytotoxicity was evaluated using the Cell Counting Kit-8 (Vazyme, Nanjing, China) following the manufacturer's instructions. This standardized protocol was subsequently applied to determine the concentration-dependent cytotoxicity of ethanol and to evaluate the protective effects of SEVs against ethanol-induced cytotoxicity (Qiu et al. 2023). Based on CCK-8 results, the experimental concentrations were determined as 100 mM for ethanol and 10 and 50 μg/mL for SEVs administration.

### Cellular internalization assay

To investigate cellular internalization of SEVs, DiL dye-labeled SEVs were prepared by incubating with DiL (Beyotime, Shanghai, China) at 37 °C for 30 min, subsequently three rounds of ultrafiltration using 100 kDa Amicon Ultra filters (Merck Millipore) to remove unbound dye. Parallel control samples were prepared by labeling PBS with equivalent DiL concentrations. Aml-12 cells were seeded into confocal dishes and allowed to adhere overnight (Li et al. 2024). Cells were then incubated with DiL-labeled SEVs for 4 h at 37 °C. Cells were fixed with 4% paraformaldehyde for 30 min, followed by three washes with PBS containing 0.1% Triton X-100. Actin cytoskeletons were stained using Actin-Tracker Green-488 for 30 min. After additional washes with Triton X-100/PBS, cell nuclei were counterstained with 4′,6-diamidino-2-phenylindole (DAPI) for 10 min. After staining and PBS washes, imaging was performed using Confocal Laser Scanning Microscope (CLSM, ZEISS, Germany).

### Animals

C57BL/6 J mice (10-week-old males) were sourced from the National Laboratory Animal Resource Center (Shanghai, China). All animal experiments were performed in strict compliance with the institutional guidelines outlined in the Guide for the Care and Use of Laboratory Animals by the Zhejiang University of Technology (Approval NO.: MGS20241203181).

### Biodistribution analysis of SEVs

To investigate the in vivo biodistribution of SEVs, SEVs were labeled with the lipophilic fluorescent dye DiR (5 μM) through 40-min incubation at 37 °C (Li et al. 2024). Unbound dye was removed via triple ultrafiltration (3,000 × g, 10 min) using 100 kDa Amicon Ultra filters. A control group received an equivalent concentration of DiR dissolved in PBS. Both DiR-labeled SEVs (DiR-SEVs) and DiR-PBS were administered to mice via oral gavage (n = 3). At predetermined intervals (1, 6, 24 h post-administration), animals were anesthetized and subjected to whole-body imaging using an IVIS Spectrum system (Em = 748 nm, Ex = 780). Following systemic imaging, major organs were excised for ex vivo fluorescence quantification using the same imaging parameters.

### Preventive effect of SEVs against alcohol-induced liver injury in mice

After a one-week acclimatization period in the animal centre, 12 mice were assigned to each of the four experimental groups. mice were randomly divided into four groups by radom number table method. All researchers performing open-field behavioral detection, histological staining, tissue imaging and biochemical quantitative analysis remained blinded to group allocation throughout data collection and statistical analysis. Predefined animal exclusion criteria were set in advance: mice with excessive weight loss, sustained poor mobility, or unexpected premature death before the experimental endpoint would be eliminated from statistical analysis. Alcohol-induced liver injury was established using the Gao-Binge model (NIAAA model) (Bertola et al. 2013). Notably, this chronic-plus-binge feeding regimen mainly recapitulates early-stage alcoholic hepatic steatosis and inflammatory injury, and cannot fully represent progressive ALD manifestations such as liver fibrosis, cirrhosis or hepatocellular carcinoma. Briefly, mice were acclimated to a liquid diet for three days and then were fed a Lieber-DeCarli diet with graded ethanol concentrations (1–4%) for four days. Subsequently, mice were fed a Lieber-DeCarli diet containing 5% ethanol or an isocaloric control diet for ten days. Food intake of ethanol-fed mice was recorded, and pair-feeding was performed to ensure equivalent dietary consumption across groups. During this period, SEVs (1.0 mg/kg/day, low dose; 10.0 mg/kg/day, high dose) were administered via oral gavage. Ethanol feeding was then discontinued for one day, and mice were provided a standard control diet. Open-field test was conducted to evaluate ethanol-induced effects on locomotor activity and neurobehavioral outcomes. Finally, a single dose of ethanol (5 g/kg) or isocaloric maltodextrin (9 g/kg) was administered via oral gavage to ethanol-fed and control groups. Mice were euthanized 9 h later, and tissues were rapidly snap-frozen and stored at − 80 °C for subsequent analysis.

### Biochemical assays

The culture supernatant of Aml-12 cells was collected to determine alanine aminotransferase (ALT) and aspartate aminotransferase (AST) levels. Intracellular catalase (CAT), superoxide dismutase (SOD), and malondialdehyde (MDA) levels were quantified using commercial assay kits. Similarly, serum and liver tissues from mice were subjected to biochemical assessments. Serum levels of ALT, AST, total bilirubin (TBil), alkaline phosphatase (AKP), gamma-glutamyl transferase (γ-GT), total cholesterol (TC), and total cholesterol (T-CHO) were measured (Nanjing Jiancheng Bioengineering Institute, Nanjing, China). Hepatic activities of CAT, SOD, glutathione peroxidase (GSH-PX), and MDA were quantified with corresponding detection kits (Servicebio, Wuhan, China). All procedures were performed in strict accordance with the instructions of manufacture.

### Histopathology analysis

The collected liver tissues were dissected into small fragments, and a subset was fixed in 4% paraformaldehyde. Fixed tissues were sequentially processed, paraffin-embedded, sectioned into 4-μm-thick slices, and stained with hematoxylin and eosin (H&E) for histological evaluation.

### Quantitative reverse-transcription PCR

Total RNA from the liver or cells was extracted using TRIzol reagent following the manufacturer’s instructions. Reverse transcription and qPCR reagents were from Vazyme. Equal-concentration RNA was reverse-transcribed into cDNA with 5 × Hiscript II qRT SuperMix II (Vazyme, Nanjing, China). qPCR was performed on a CFX Connect Real-Time System (BIO-RAD) using 2 × ChamQ Universal SYBR. GAPDH and β-actin were used as housekeeping genes to analyze inflammation- and oxidative stress-related gene expression (Fang et al. 2025). Primer sequences are in the Supplementary Table S2.

### Western blot

Liver tissues were homogenized in RIPA buffer (Serve, Wuhan, China) supplemented with protease and phosphatase inhibitors (Serve, Wuhan, China). Protein concentrations were quantified using a BCA assay. Equal amounts of protein lysates were separated on 10% SDS-PAGE, transferred to polyvinylidene difluoride (PVDF) membranes, and blocked. Membranes were incubated with primary antibodies overnight, followed by horseradish peroxidase (HRP)-conjugated secondary antibodies (Huabio, Hangzhou, China). Protein signals were visualized using an enhanced chemiluminescence (ECL) substrate kit (Serve, Wuhan, China), and the intensity of the signals was quantified using ImageJ software. The antibody information used in the Western blot is shown below: His Tag (Daigebio, #db11052), anti-heme oxygenase1 (HO-1) (Huabio, # ER1802-73), and anti-Nrf2 (Huabio, #HA721432).

### Statistical analysis

Statistical analyses were conducted using GraphPad Prism software (version 10.1.2). Prior to intergroup comparison, Shapiro–Wilk test was applied to verify data normality, and Brown-Forsythe test was used to assess homogeneity of variance. All quantitative datasets including hepatic biochemical indicators, mRNA relative expression, and Western blot grayscale quantification (Fig. 6A–H) met the criteria for parametric analysis. All experiments were performed with defined biological replicates (exact group n values are indicated within each figure legend) and independent technical replicates to ensure reproducibility. Intergroup comparisons were performed using one-way analysis of variance (ANOVA) with Tukey's post hoc test for multiple comparisons. A probability value of *P* < 0.05 was considered statistically significant for all analyses.

## Results

### Engineered surface display of CotC-SOD-His fusion protein on the EVs

**Fig. 1:**
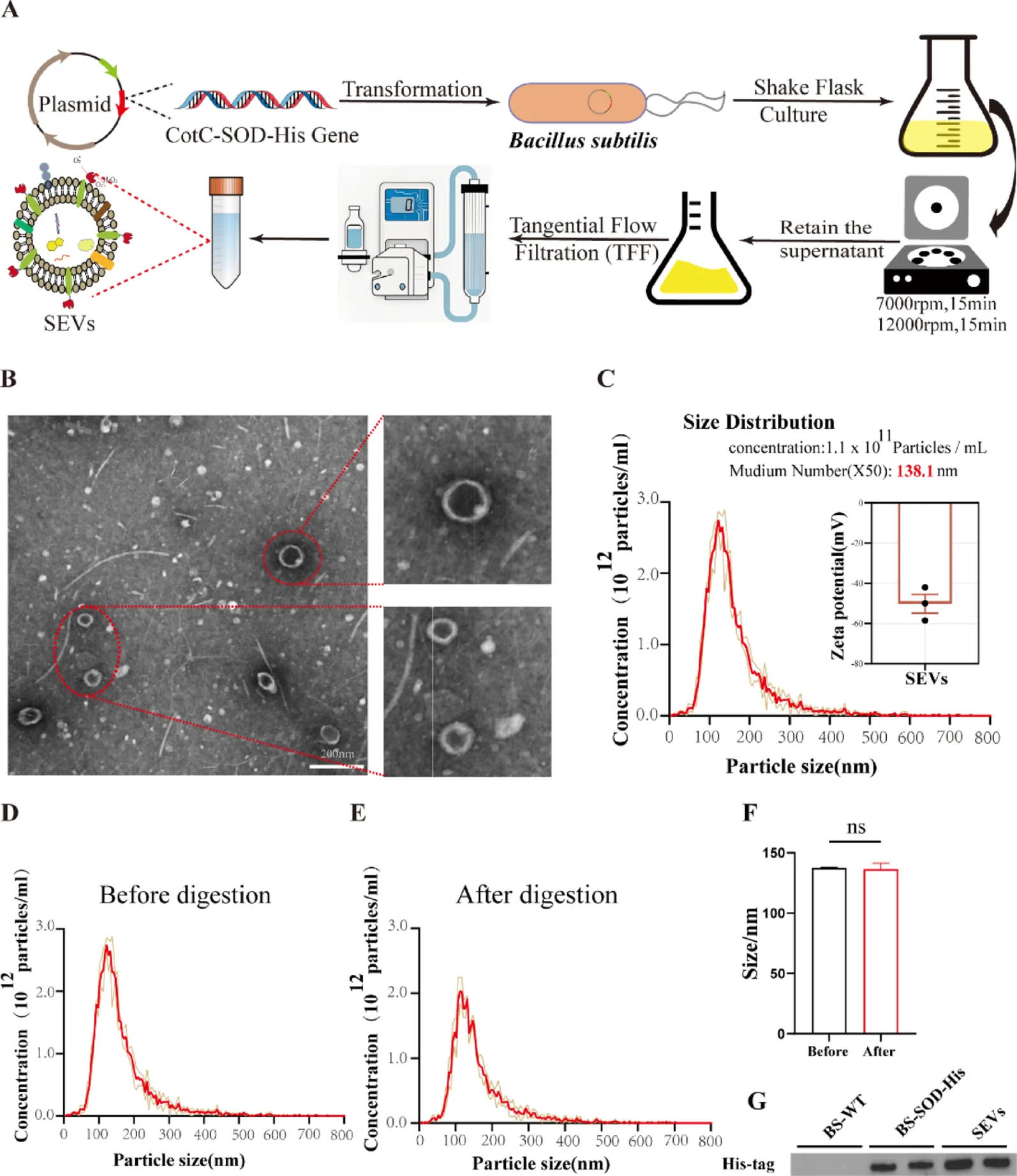
Preparation and characterization of engineered SEVs. **A** Engineering of BS168 and EV isolation via TFF system. **B** TEM morphology of SEVs (scale bar: 200 nm). **C** NTA size distribution and zeta potential of SEVs. **D**–**E** Size changes of SEVs before and after simulated digestive fluid treatment. **F** Quantitative analysis of SEVs particle size. **G** Western blot detection of His-Flag expression

Building upon surface display methodology, we engineered EVs from *Bacillus subtilis*168 by genetically anchoring the exogenous CotC-SOD-His fusion protein within the EVs (Mauriello et al. 2004; Wang et al. 2023). As schematically illustrated in Fig. 1A, the genes encoding the spore coat protein CotC and SOD were cloned into plasmid pMA09, followed by the insertion of a His-tag sequence further to verify the existence of SOD in the derived EVs. The recombinant plasmid was transformed into *Bacillus subtilis* 168, and SEVs were mass-produced using TFF. Under the same experimental conditions, wild-type *Bacillus subtilis* 168 was utilized as a negative 122and EVs derived from wild-type *Bacillus subtilis* 168 were isolated using TFF system and termed WEVs. Structural characterization via TEM confirmed the cup-shaped morphology and bilayer membrane architecture of SEVs. At the same time, NTA analysis revealed an average diameter of 138.1 nm, a yield of 1.1 × 10^11^ particles/mL of SEVs, and the surface potential of SEVs was -49.6 mV, indicating its stability (Fig. 1B and 1C). Moreover, after incubation at 4 °C and 37 °C for 24 h, the particle numbers of SEVs were 8.1 × 10⁹ particles/mL and 7.3 × 10⁹ particles/mL respectively, while their particle size and zeta potential showed no significant alterations, further verifying the favorable physicochemical stability of SEVs under common storage conditions (Figure S3). Western blot analysis targeting the His-tag demonstrated robust expression of the CotC-SOD-His fusion protein in both lysates of recombinant *Bacillus subtilis* 168 (BS168-pMA09-CotC-SOD-His) and SEVs, with significantly stronger His-tag signals in SEVs compared to bacterial lysates at equivalent protein concentrations. No His-tag signal was detected in lysates of wild-type *Bacillus subtilis* 168, confirming the specificity of the surface display system (Fig. 1G).

### SEVs exhibit high resistance to gastrointestinal digestion

Following oral gavage administration, SEVs initially traversed the gastrointestinal tract prior to systemic dissemination to target organs. This necessitates a rigorous evaluation of their stability against gastrointestinal digestive fluids. To simulate physiological digestion, we employed an ex vivo digestion model from established protocols. NTA revealed no statistically significant alterations in the mean particle diameter of SEVs, though a marginal reduction in particle yield was observed (Fig. 1D-F). Meanwhile, SOD enzymatic activity assays demonstrated that the SOD activity carried by SEVs did not exhibit a significant decrease before and after exposure to simulated gastrointestinal fluids; additionally, Western blot analysis confirmed the structural stability of the CotC-SOD fusion protein in samples pre- and post-digestion (Figure S4). These data demonstrate that SEVs maintain structural integrity under gastrointestinal conditions, thereby retaining their capacity to enter systemic circulation, accumulate in distal organs, and mediate functional effects.

### SEVs alleviate lipid accumulation in alcohol-induced hepatocyte injury

**Fig. 2:**
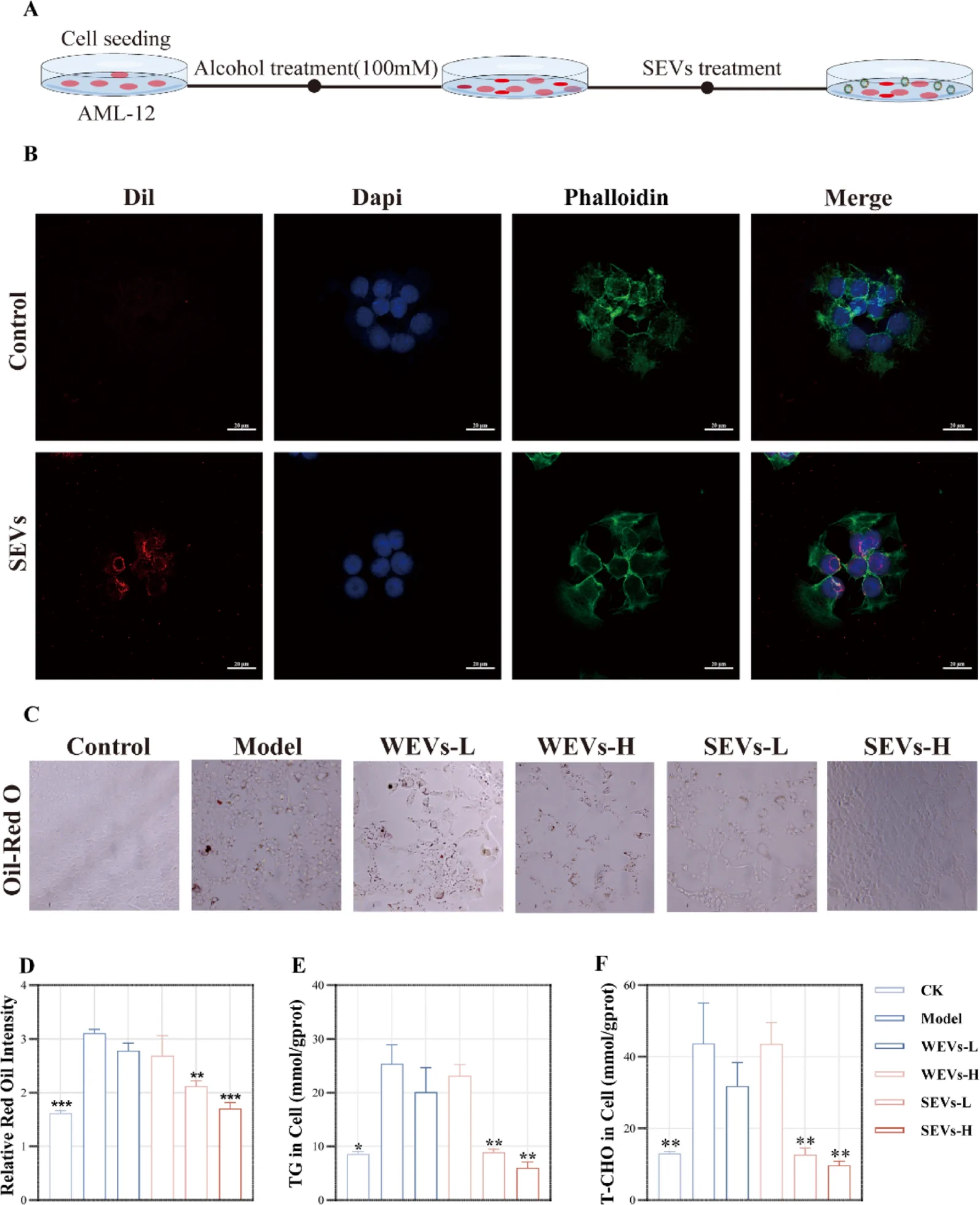
SEVs ameliorate lipid accumulation in alcohol-injured AML-12 cells. **A** Schematic timeline of alcohol injury and SEVs treatment in AML-12 cells. **B** Cellular internalization of SEVs (scale bar: 20 μm). **C** Oil Red O staining and **D** quantitative analysis of intracellular lipid deposition. **E**–**F** Contents of TG and T-CHO in cells. Data are mean ± SEM; *P < 0.05, **P < 0.01, ***P < 0.001, ****P < 0.0001

In our experimental design, SEVs were engineered to carry SOD, thereby conferring potential antioxidant activity (Ran et al. 2024; Zhou et al. 2022). To further validate the protective efficacy of SEVs, we established an alcohol-induced injury model in Aml-12 hepatocytes to investigate the reparative effects of SEVs. The optimal alcohol modeling concentration and SEVs dosing regimen were determined using CCK-8 assays. CCK-8 results revealed that exposure to 100 mM alcohol significantly reduced the viability of cells to 58 percent, whereas SEVs exhibited no apparent cytotoxicity toward Aml-12 cells (Tian et al. 2014). Notably, SEVs significantly restored the viability of Aml-12 cells treated with 100 mM alcohol (Figure S1). As illustrated in Fig. 2A, we selected 100 mM alcohol for modeling and administered SEVs at low (10 μg/mL) and high (50 μg/mL) doses for subsequent experiments, with WEVs serving as controls. To evaluate the uptake of SEVs by Aml-12 cells, DiL-labeled SEVs were co-incubated with the cells for 4 h, and their intracellular localization was visualized using CLSM. The results demonstrated that red fluorescence was clearly observed within Aml-12 cells after 4 h of co-incubation, whereas no fluorescence was detected in the negative control group (Fig. 2B). Oil Red O staining demonstrated that alcohol-treated hepatocytes exhibited pronounced accumulation of red lipid droplets. Both low- and high-dose WEVs groups displayed similar lipid deposition patterns, whereas SEVs intervention markedly reduced lipid droplet aggregation in a dose-dependent manner (Fig. 2C). Quantitative analysis of Oil Red O staining corroborated these findings indicating that SEVs effectively alleviated alcohol-induced hepatic steatosis (Fig. 2D). Biochemical assays further confirmed that SEVs significantly reduced intracellular TG and T-CHO levels in alcohol-treated hepatocytes (Fig. 2E and F). Collectively, these results demonstrate that SEVs, which are efficiently internalized by hepatocytes, attenuate alcohol-induced lipid accumulation in these cells.

### SEVs alleviate alcohol-induced hepatic cell injury by regulating oxidative stress

**Fig. 3:**
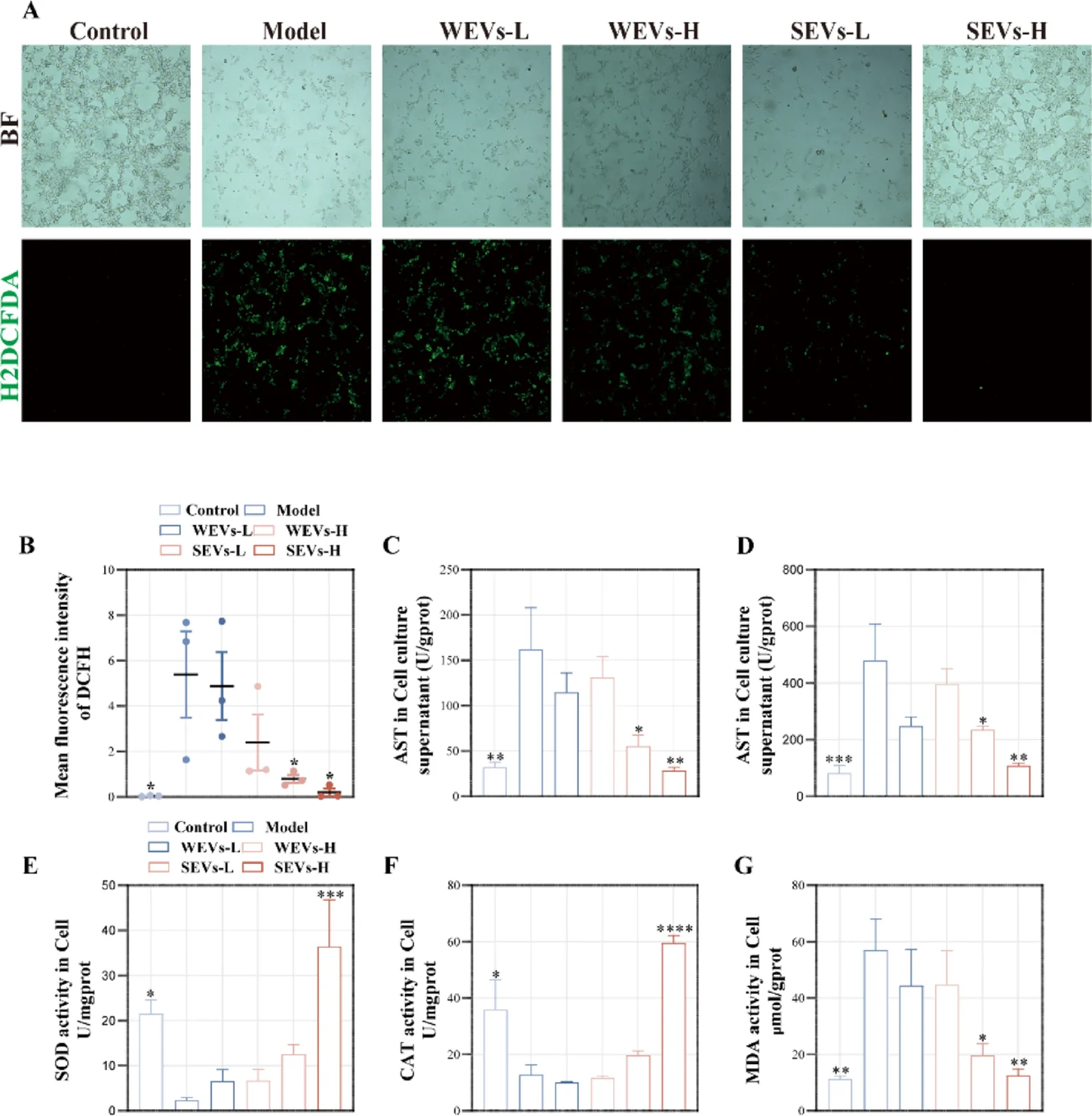
SEVs relieve oxidative stress in alcohol-damaged hepatocytes. **A** Cellular ROS staining and **B** quantitative analysis. **C**–**D** Levels of AST and ALT in cell culture supernatant. **E**–**G** Contents of SOD, CAT and MDA in cells. Data are mean ± SEM; *P < 0.05, **P < 0.01, ***P < 0.001, ****P < 0.0001

Oxidative stress is a pivotal mechanism underlying alcohol-induced hepatocyte injury (Li et al. 2015; Park et al. 2023; Salete-Granado et al. 2023; Yang et al. 2024). To evaluate whether SEVs ameliorate this process, intracellular ROS levels were assessed. Alcohol treatment markedly increased ROS fluorescence intensity, which was moderately reduced by high-dose WEVs, though not statistically significant. In contrast, SEVs intervention significantly suppressed ROS generation and alleviated oxidative stress, with high-dose SEVs restoring ROS levels to near-normal values. Quantitative analysis of ROS further confirmed this trend (Fig. 3A and B). Biochemical analyses revealed that SEVs robustly enhanced intracellular SOD and CAT activities while reducing MDA levels, indicating that SEVs bolster the cellular antioxidant defense system to counteract alcohol-induced oxidative damage (Fig. 3E–G). Furthermore, alcohol exposure markedly elevated ALT and AST levels in cell culture supernatants, reflecting hepatocellular injury (Fig. 3C and D) (Ren et al. 2019; Wang et al. 2015). SEVs treatment significantly reversed these alterations, whereas WEVs showed no effect, underscoring the unique reparative capacity of SEVs against alcohol-induced hepatocyte damage. In conclusion, our findings show that SEVs reduce alcohol-induced hepatocyte damage by boosting antioxidant capacity, thereby safeguarding against alcohol-related liver dysfunction.

### SEVs alleviate anxiety-like behaviors and improve motor activity in ALD Mice

**Fig. 4:**
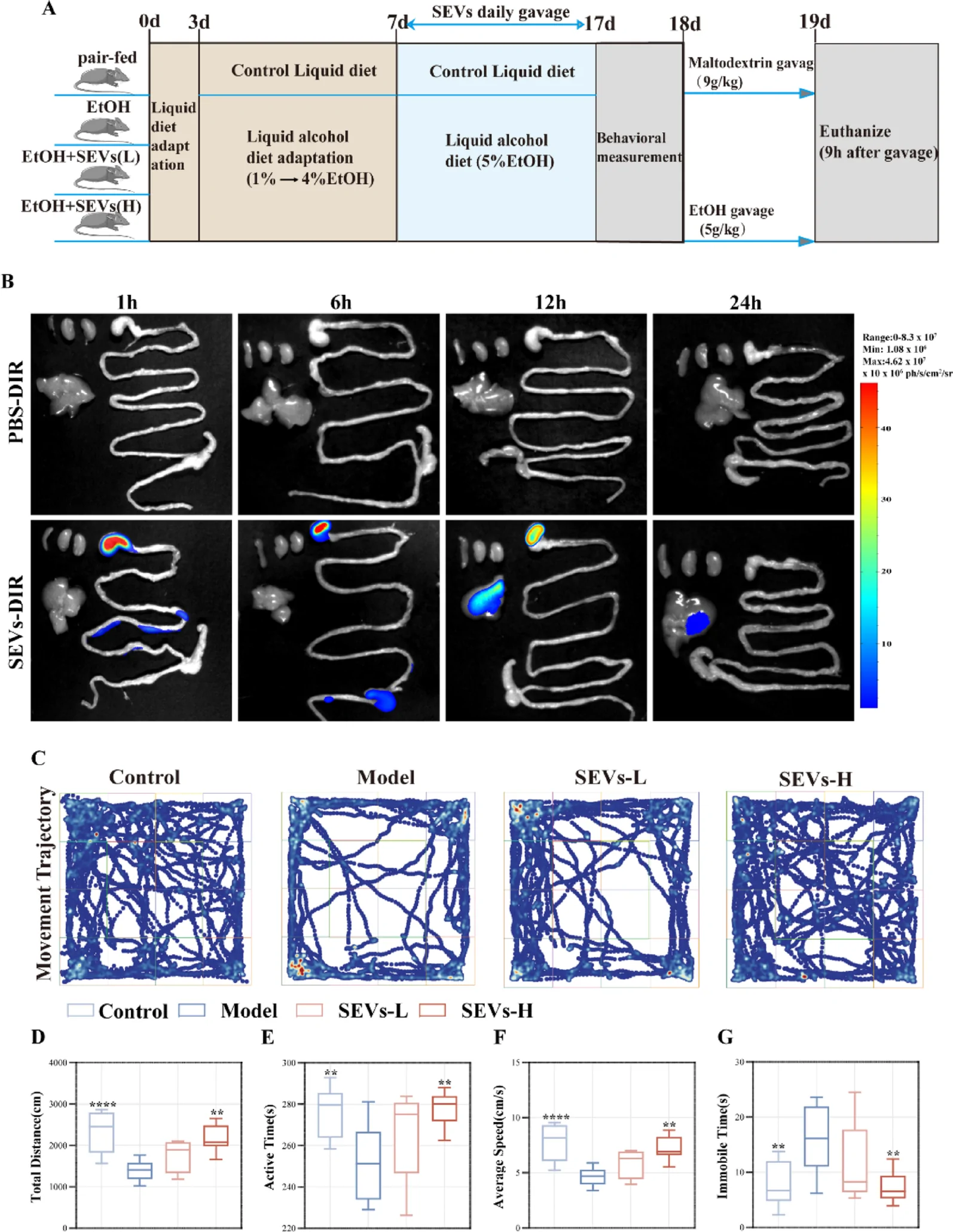
In vivo preventive protective evaluation of oral SEVs in alcoholic liver injury mice. **A** Schematic protocol of alcoholic liver injury model establishment and SEVs administration. **B** Organ biodistribution of SEVs at 1, 6, 12 and 24 h after oral delivery. **C**–**G** Open-field behavioral trajectories and quantitative analysis of total distance, active time, average speed and immobile time. Data are mean ± SEM

Based on the promising restorative effects of SEVs in alcoholic hepatocyte cellular models, we further evaluated the hepatoprotective potential of SEVs in mice using the Gao-Binge ethanol-induced liver injury model (10-day chronic ethanol diet followed by a single binge ethanol gavage) with experimental design illustrated in Fig. 4A. The in vivo distribution and tissue-targeting specificity of SEVs are critical for their therapeutic efficacy. To investigate this, mice were administered DIR-labeled SEVs via oral gavage, and whole-body fluorescence imaging was performed at different time points (1, 6, 12, 24 h) using an IVIS imaging system. After imaging, mice were euthanized, and fluorescence signals in harvested tissues were quantified. The imaging results revealed that SEVs predominantly accumulated in the gastrointestinal tract at both 1 h and 6 h post-administration. By 12 h, fluorescence signals were detected in the liver, and at 24 h, signals in other tissues had diminished, while residual fluorescence persisted in the liver (Figs. 4B and S2). These findings indicate that SEVs undergo systemic circulation and selectively enrich in the liver, where they exhibit prolonged retention compared to other tissues, suggesting their potential to exert sustained functional effects in hepatic tissue.

To assess the impact of ethanol consumption on behavioral phenotypes, open field tests were conducted after 24-h alcohol abstinence. Movement trajectory heatmaps revealed that alcohol-fed mice exhibited significantly reduced exploration time in the central zone with corner-preference behavior, which was remarkably reversed by SEVs administration (Fig. 4C). Quantitative analysis demonstrated that alcohol-fed mice showed significant decreases in total distance, active time, and average speed, along with increased immobile time (Fig. 4D-G). These behavioral impairments were substantially ameliorated by SEVs intervention, particularly evident in the high-dose group. These findings collectively indicate that chronic ethanol exposure induces anxiety-like behaviors and impairs spontaneous locomotor activity in mice, while SEVs treatment effectively alleviates anxiety-related manifestations and restores motor function.

### SEVs attenuate alcohol-induced liver injury and steatosis in mice

**Fig. 5:**
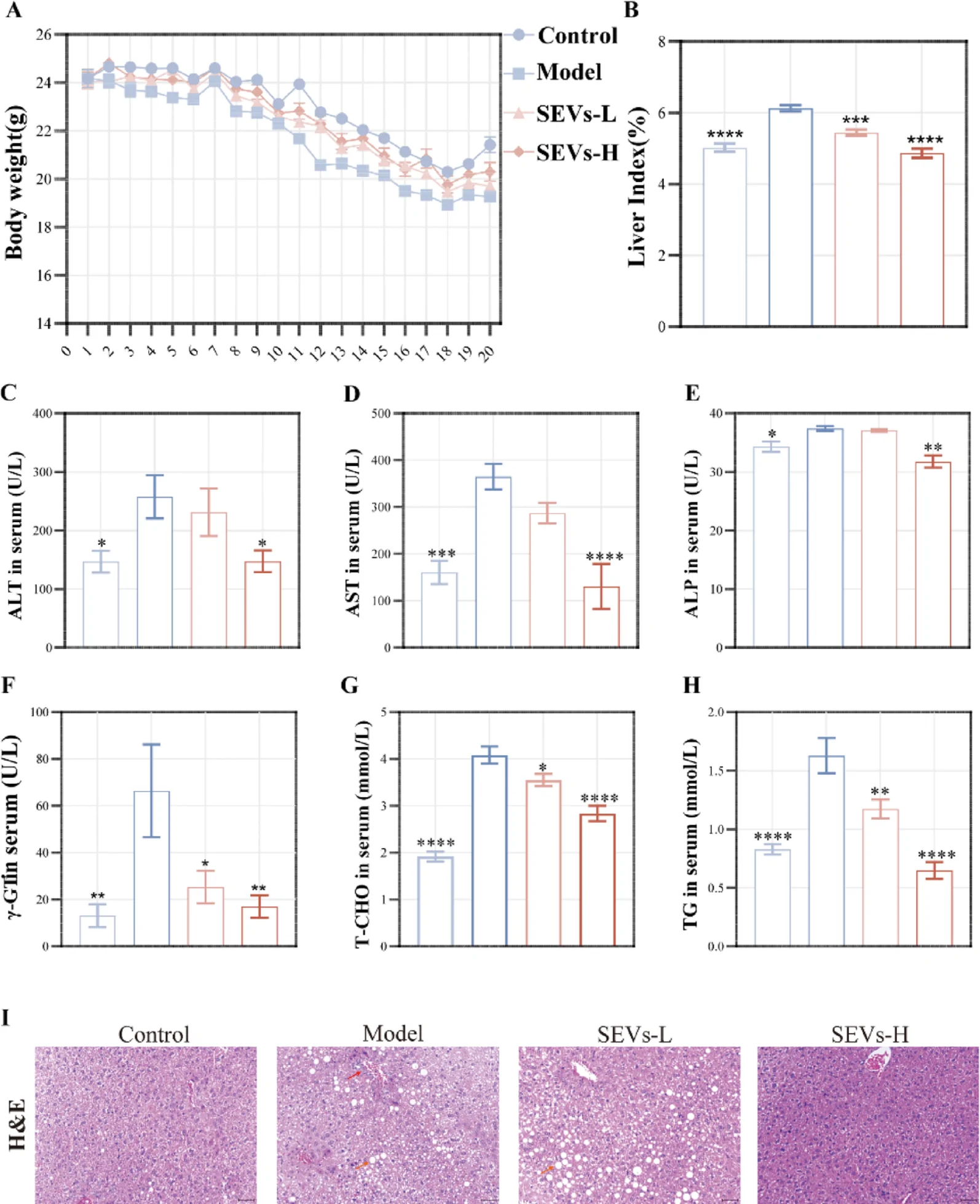
SEVs improve liver function and lipid metabolism in alcoholic liver injury mice. **A** Body weight changes of mice. **B** Liver index of each group. **C**–**F** Serum levels of ALT, AST, ALP and γ-GT. **G**–**H** Serum TC and T-CHO contents. **I** Liver H&E staining. Data are mean ± SEM; *P < 0.05, **P < 0.01, ***P < 0.001, ****P < 0.0001

To further explore the protective effects of SEVs on alcohol-induced liver injury, we first monitored the body weight dynamics and liver weight alterations in mice. As shown in Fig. 5A, although alcohol-fed mice exhibited reduced body weight compared to pair-fed controls, this difference did not reach statistical significance. Notably, the liver index (liver-to-body weight ratio) increased significantly in alcohol-fed mice relative to controls. SEVs treatment effectively reduced this alcohol-induced elevation in a dose-dependent manner, suggesting that SEVs ameliorate pathological hepatic changes caused by alcohol consumption (Fig. 5B). To further elucidate the liver-protective potential of SEVs, we next examined the serum biomarkers related to liver function and lipid metabolism. Serum biomarkers of liver injury, including ALT, AST, ALP, and γ-GT, were markedly elevated in alcohol-fed mice (Fig. 5C-F). Concurrently, serum TG and T-CHO levels were significantly increased, indicating disrupted lipid metabolism and hepatic dysfunction (Fig. 5G and H). SEVs administration substantially mitigated the alcohol-induced increases in these liver injury markers and lipid profiles, demonstrating their hepato-protective effects.

Histopathological analysis confirmed these biochemical findings. Alcohol-fed mice showed extensive hepatic lipid vacuolation and infiltration of inflammatory cells, while high-dose SEVs treatment notably decreased lipid droplet size and inflammatory foci (Fig. 5I). Previous in vivo imaging verified the hepatic targeting of SEVs. Overall, these findings indicate that SEVs help reduce alcohol-induced hepatocyte damage, steatosis, and hepatic inflammation, possibly by modulating liver-specific signalling pathways.

### Correlation between Nrf2/HO-1 upregulation and relieved hepatic oxidative/inflammatory injury in SEVs-treated alcohol-exposed mice

**Fig. 6:**
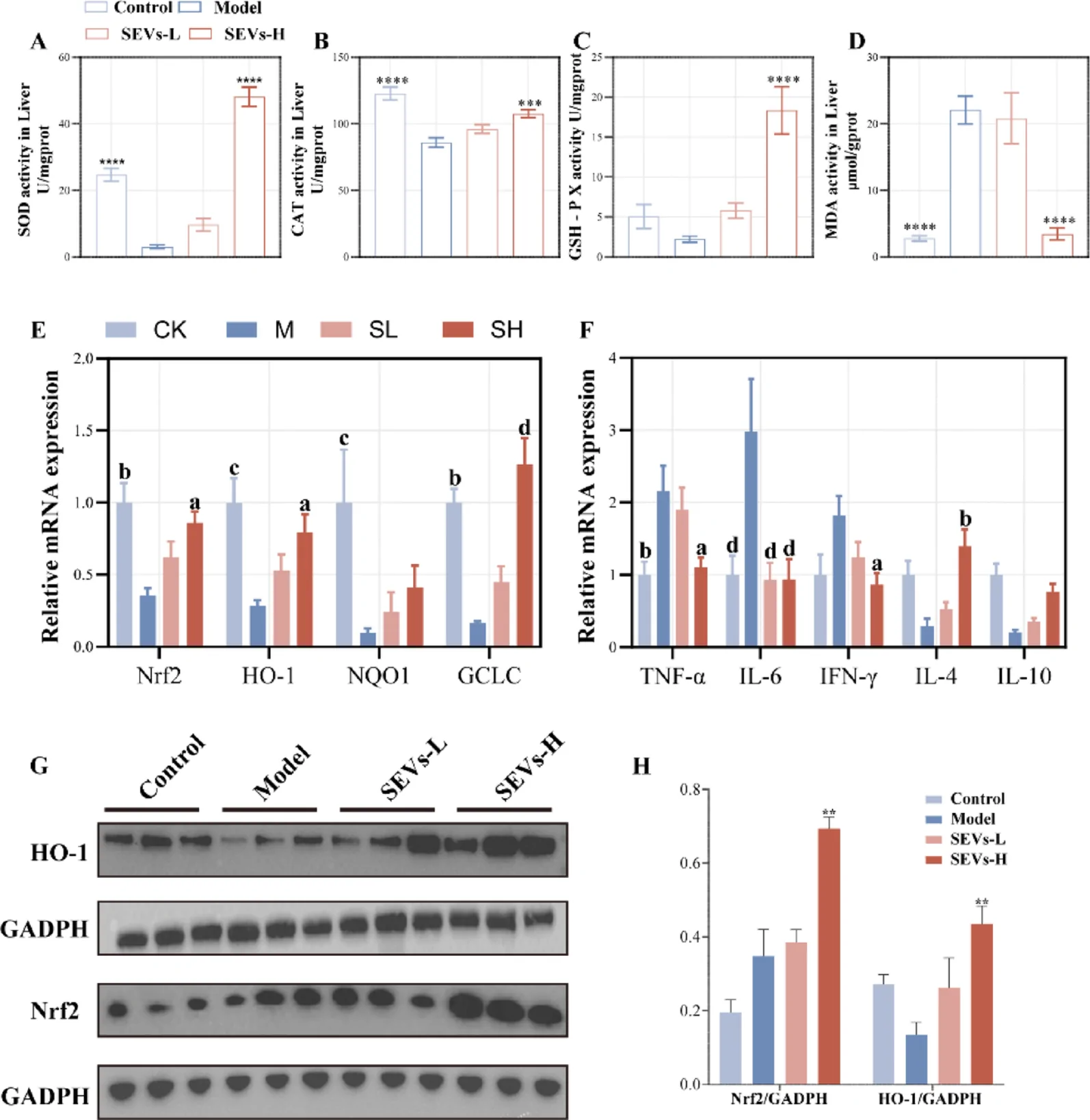
Antioxidant and anti-inflammatory mechanisms of SEVs via Nrf2/HO-1 pathway. **A**–**D** Hepatic levels of SOD, CAT, GSH-PX and MDA. **E** mRNA expression of Nrf2, HO-1, NQO1 and GCLC. **F** Inflammatory cytokine mRNA levels of TNF-α, IL-6, IFN-γ, IL-4 and IL-10. **G**–**H** Protein expression and quantitative analysis of Nrf2 and HO-1. Data are mean ± SEM; *P < 0.05, **P < 0.01, ***P < 0.001, ****P < 0.0001

Given the central role of oxidative stress in alcohol-related liver pathology, we focused on the Nrf2/HO-1 axis, a master regulator of cellular antioxidant defenses (Li et al. 2020; Niu et al. 2024; Sun et al. 2018; Wu et al. 2025). Alcohol-fed mice displayed severe hepatic oxidative injury, with significantly reduced activities of antioxidant enzymes (SOD, CAT, and GSH-PX) and elevated MDA levels compared to control mice. SEVs treatment dose-dependently restored antioxidant enzyme activities and suppressed lipid peroxidation (Fig. 6A-D). To explore molecular alterations associated with SEVs-mediated liver protection, we examined the Nrf2/HO-1 pathway. Alcohol exposure markedly downregulated mRNA expression of *Nrf2* and its downstream targets (*HO-1, NQO1, and GCLC*). In contrast, SEVs treatment significantly upregulated the transcription of these genes (Fig. 6E). This divergent trend between Nrf2 mRNA and protein may be partly attributed to post-translational regulation of the Keap1-Nrf2 axis. Alcohol-triggered oxidative stress could slow Nrf2 protein turnover without altering gene transcription, and accumulated nuclear Nrf2 might trigger mild negative feedback to restrain Nrf2 mRNA synthesis. The asynchronous timing of transcriptional and protein responses may also contribute to this inconsistency, which has been observed in previous ALD studies. Western blot and quantitative analysis revealed that compared with control diet-fed mice, alcohol diet-fed mice exhibited a compensatory upregulation of Nrf2 protein expression in the liver, though this difference did not reach statistical significance. Moreover, administration of SEVs significantly elevated the protein expression levels of both Nrf2 and HO-1 in mouse livers, with the high-dose SEVs group demonstrating particularly marked enhancement in Nrf2 and HO-1 expression (Fig. 6G and H). Notably, the above changes in gene and protein expression only represent correlative molecular alterations following SEVs intervention. Without genetic knockout or silencing assays targeting Nrf2 or HO-1, we cannot confirm that activation of the Nrf2/HO-1 axis is the direct causal factor responsible for the hepatoprotective effect of SEVs.

These correlated molecular phenotypes merely suggest that elevated Nrf2/HO-1 signaling may be one potential contributor to the relief of hepatic oxidative stress in alcohol-exposed mice treated with SEVs, rather than a fully validated definite functional mechanism. Inflammatory responses represent yet another crucial pathogenic factor contributing to alcohol-related liver injury. The activation of the Nrf2/HO-1 pathway effectively curbs inflammation via a multitude of mechanisms (Geertsema et al. 2023; Leon-Icaza et al. 2025; O’Rourke et al. 2024; Saha et al. 2020). To further characterize the inflammatory molecular profile after SEVs administration, we performed qPCR analysis of hepatic inflammatory cytokines in mice. The results demonstrated that SEVs markedly attenuated alcohol-induced hepatic inflammation, as evidenced by significantly reduced mRNA levels of pro-inflammatory cytokines (*TNF-α,IL-6,and IFN-γ*) and upregulated expression of anti-inflammatory mediators (*IL-4, IL-10*) (Fig. 6F). In summary, our molecular detection data show a strong correlation between SEVs treatment and the upregulation of the Nrf2/HO-1 antioxidant pathway, accompanied by suppressed hepatic pro-inflammatory gene expression. We speculate that Nrf2/HO-1 signaling may participate in the hepatoprotective effects of SEVs by improving intracellular antioxidant capacity and restraining inflammatory cascades. Nevertheless, targeted genetic interference experiments are still required to clarify the direct causal contribution of this pathway to the therapeutic efficacy of SEVs.

## Discussion

In this study, we developed an orally administrable spore-derived extracellular vesicle platform displaying superoxide dismutase (SEV-SOD) and demonstrated its preventive hepatoprotective efficacy against alcoholic liver disease (ALD) through activation of the Nrf2/HO-1 antioxidant pathway. This platform combines the advantages of Bacillus subtilis spore surface display technology—well-established for stable heterologous protein expression—with scalable tangential flow filtration (TFF) production, addressing a key bottleneck in the clinical translation of bacterial extracellular vesicle (BEV)-based therapeutics.

SEV characterization confirmed the suitability of this platform for oral delivery. The vesicles exhibited a cup-shaped morphology with an average diameter of 138.1 nm and a zeta potential of − 49.6 mV. Their physicochemical properties remained stable at both 4 °C and 37 °C, and SEVs withstood simulated gastric and intestinal fluids without significant degradation. Together, these characteristics address two persistent challenges in BEV-based therapy: storage stability and gastrointestinal barrier survival. While previous studies have leveraged BEVs for drug delivery, scalable production and oral bioavailability have remained limiting factors (De Langhe et al., 2024; Zheng et al., 2024). Our results suggest that the spore display–TFF workflow may offer a practical solution to both limitations.

Orally administered SEVs exhibited preferential accumulation in the liver. After gavage, DiR-labeled SEVs shifted from predominant gastrointestinal distribution (1–6 h) to selective hepatic enrichment by 12 h, and remained detectable exclusively in the liver at 24 h. This prolonged retention profile aligns with reports for MSC-derived and hepatocyte-derived EVs (Du et al., 2025; Wang et al., 2023b; Yang et al., 2025), suggesting a common, size- and surface-dependent liver-targeting mechanism among diverse EV subtypes. The observed enrichment pattern is consistent with trans-intestinal translocation via portal circulation, followed by hepatocyte uptake mediated by EV surface ligands and the negatively charged membrane. Notably, ALD-associated inflammation may further potentiate EV internalization, as damaged hepatocytes upregulate scavenger receptors and endocytic activity. Nevertheless, the precise molecular determinants of this hepatic tropism—and whether they can be enhanced through surface engineering—require dedicated investigation.

Compared with existing SOD-based therapeutic strategies, SEV-SOD offers several distinct advantages. Free SOD is limited by rapid clearance and gastrointestinal instability; chemically modified SOD often compromises catalytic activity; and synthetic nanoparticle carriers involve complex fabrication and suboptimal biocompatibility. SEV-SOD circumvents these drawbacks through facile biosynthetic production, favorable safety profile, and most importantly, the presence of endogenous antioxidant proteins within SEVs that can act synergistically with surface-displayed SOD. This multi-component antioxidant capacity represents a qualitative advantage over single-molecule SOD formulations and may be particularly relevant for ALD, where oxidative stress involves multiple ROS species and subcellular compartments.

In vivo, SEV administration reversed alcohol-induced behavioral and biochemical abnormalities. In the Gao-Binge (NIAAA) mouse model, SEV treatment attenuated anxiety-like behaviors and locomotor deficits, improved serum markers of liver injury (ALT, AST), and reduced hepatic lipid accumulation. Correspondingly, in ethanol-treated Aml-12 hepatocytes, SEVs relieved oxidative stress and normalized lipid metabolism. These findings indicate that SEV-SOD confers functional protection at both the hepatic and systemic levels, the latter being particularly noteworthy given that alcohol-induced neurobehavioral impairment is often refractory to hepatocentric interventions.

Mechanistically, the protective effects of SEVs were mediated through the Nrf2/HO-1 signaling axis. SEV treatment upregulated Nrf2 and HO-1 expression at both mRNA and protein levels and suppressed pro-inflammatory cytokine expression in liver tissue. Given the central role of Nrf2/HO-1 in orchestrating antioxidant and anti-inflammatory responses (Guo et al., 2025b; Li et al., 2022), these results provide a plausible mechanism for the multi-faceted protection observed. However, a key open question is how SEVs engage this pathway from the extracellular space. Whether the activation is triggered by the relief of ROS burden (feedback derepression), by direct EV–receptor interactions on hepatocytes, or by EV-delivered signaling molecules remains unresolved and merits further investigation through systematic loss-of-function approaches.

Several limitations should be acknowledged. First, the current study did not include SEVs derived from empty-plasmid transformants as a control, leaving it unclear what fraction of the therapeutic effect is attributable to SOD display versus the EV scaffold itself. Future experiments incorporating vector-only SEVs and SOD-deficient mutants will be necessary to deconvolve these contributions. Second, while Nrf2/HO-1 activation was clearly demonstrated, the upstream signaling events and potential crosstalk with other pathways (e.g., autophagy, ferroptosis) were not explored. Third, the protective effect evaluation was confined to a single mouse model and an acute-on-chronic alcohol challenge; chronic feeding models and longer treatment windows would better assess translational relevance. Fourth, the long-term safety of repeated SEV administration—particularly regarding immunogenicity and off-target effects—has not been evaluated and warrants careful assessment before clinical consideration.

In summary, SEV-SOD combines the scalability of bacterial spore display technology, the oral bioavailability of BEVs, and the multi-component antioxidant capacity of a nature-derived delivery vehicle to achieve robust protection against ALD through Nrf2/HO-1 pathway activation. The present findings establish a foundation for further development of spore-derived EVs as a platform for gastrointestinal–liver axis protective interventions.

## Conclusion

In conclusion, this study engineered *Bacillus subtilis* 168 by anchoring the SOD protein onto the membrane surface, thus obtaining EVs containing SOD. Characterizations showed that SEVs had a stable structure, resisted gastrointestinal digestion, and targeted the liver. In vitro, SEVs mitigated alcohol-induced hepatocyte injury by regulating lipid metabolism and enhancing antioxidant capacity, reducing lipid accumulation and ROS generation. In ALD mouse model, SEVs alleviated liver damage, steatosis, and reversed alcohol-induced behavioral impairments. Mechanistically, SEVs activated the Nrf2/HO-1 pathway, enhancing antioxidant defenses and suppressing inflammation. These findings highlighted SEVs as a promising preventive hepatoprotective strategy to block the progression of alcohol-related liver diseases.

## Supplementary Information

Below is the link to the electronic supplementary material.

Supplementary Material 1

## Data Availability

All data produced or analyzed and materials for this study are available in this article and its additional information files.
