# Supplementary material for: Bacillus subtilis extracellular vesicles surface-displaying superoxide dismutase attenuate alcoholic liver injury by activating the Nrf2/HO-1 axis
Source: Bioresour Bioprocess. 2026 Sep 12;13(1):132. doi: 10.1186/s40643-026-01112-6 (PMC13569742; doi:10.1186/s40643-026-01112-6)
Supplement: Supplementary file 1 [file 40643_2026_1112_MOESM1_ESM.docx]

***­­Bacillus subtilis* extracellular vesicles surface-displaying superoxide dismutase attenuate alcoholic liver injury by activating the Nrf2/HO-1 axis**

Baoxian Li^1,2#^, Jiali Chen^1,2#^, Chaozhi Wei^2^, You Wei^1,2^, Chunqiang Pan^1,2^, Tao Liu^2^, Qingchi Wang^2^, Shiyu Li^2^, Yao Zhao^2^, Mengyu Zhang^2^, Chenfan Sun^4^, Chengran Guan^5^, [Domenico Nuzzo](https://www.sciencedirect.com/author/13008225400/domenico-nuzzo)^6^, Jintao Cheng^2^*, Yuanxiang Jin^1,2^*, Guiling Yang^2,3^*

^1^ College of Biotechnology and Bioengineering, Zhejiang University of Technology, Hangzhou, 310032, China

^2^ Xianghu Laboratory, Hangzhou, 311231, China

^3^ State Key laboratory for Managing Biotic and Chemical threats to the Quality and safety of agro-products, laboratory (Hangzhou) for risk assessment of agricultural Products of Ministry of agriculture, institute of agro-product safety and nutrition, Zhejiang academy of agricultural sciences, Hangzhou, 310021, China

^4^ Zhejiang Key Laboratory of Public Health Detection and Pathogenesis Research，Department of Microbiology, Zhejiang Provincial Center for Disease Control and Prevention, Hangzhou, 310051, China

^5^ Key Lab of Dairy Biotechnology and Safety Control, College of Food Science and Engineering, Yangzhou University, Jiangsu 225127, China

^6^ Istituto per la Ricerca e l’Innovazione Biomedica, CNR, via U. La Malfa 153, Palermo 90146, Italy

^#^These authors contributed equally to this work

**Corresponding authors**

Guiling Yang ([guilingchina2008@163.com](mailto:guilingchina2008@163.com))

Yuanxiang Jin (jinyx@zjut.edu.cn)

Jintao Cheng ([jintaocheng@zju.edu.cn](mailto:jintaocheng@zju.edu.cn))

Present address

Xianghu laboratory Chuangye Valley Building, No. 168, Gengwen Road, Xiaoshan District, Hangzhou, China 311231

**Supplementary Table S1.** A list of plasmids constructed in this study.

| Name | Description | Source |
| --- | --- | --- |
| pMA09-CotC-SOD | *B. subtilis* pMA09; HpaⅡ; AmpR; Kan | Preserved in our lab |
| pMA09-CotC-SOD-6xHis | *B. subtilis* pMA09; HpaⅡ; AmpR; Kan | Preserved in our lab |

Note: Amp, Ampicillin; Kan, Kanamycin

**Supplementary Table S2.** A list of primers used in this study.

| Oligo | Sequences (5’-3’) |
| --- | --- |
| His-F | CATCATCATCACCACCACTAAAGTCGAGCTCAAGCTAGCTTGG |
| His-R | GTGGTGGTGATGATGATGTCTCGTCGCATCAAAACGC |
| pMA09-Ter-check-R | CAGCTTGCTTTCGAGGTGAATTTC |
| Nrf2-F | TGGATGTGCTGCCCTAGTTA |
| Nrf2-R | TGGTGATGGAATGTAGGTGAA |
| HO-1-F | CAGGAGGGCAGAAGATTGAG |
| HO-1-R | AGAGGAGGCGGTCTTAGC |
| NQO1-F | GGCAGAAGAGCACTGATCGA |
| NQO1-R | TCCTGCAGCGAAGTGATATT |
| GCLC-F | CCAGCTCCATGATGTGTCCT |
| GCLC-R | TCAGCATGGTGGTGAAGAAG |
| β-actin-F | GGCTGTATTCCCCTCCATCG |
| β-actin-R | CCAGTTGGTAACAATGCCATGT |
| TNF-α-F | CAGGCGGTGCCTATGTCTC |
| TNF-α-R | CGATCACCCCGAAGTTCAGTAG |
| IFN-γ-F | ATGAACGCTACACACTGCATC |
| IFN-γ-R | CCATCCTTTTGCCAGTTCCTC |
| IL-4-F | ACAGGAGAAGGGACGCCAT |
| IL-4-R | GAAGCCCTACAGACGAGCTCA |
| IL-6-F | TAGTCCTTCCTACCCCAATTTCC |
| IL-6-R | TTGGTCCTTAGCCACTCCTTC |
| IL-10-F | GCTCTTACTGACTGGCATGAG |
| IL-10-R | CGCAGCTCTAGGAGCATGTG |

**Supplementary Table S3.** A list of gene coding sequences used in this study.

| Gene | Coding sequences (5’-3’) |
| --- | --- |
| *CotC* | ATGGGTTATTACAAAAAATACAAAGAAGAGTATTATACGGT  CAAAAAAACGTATTATAAGAAGTATTACGAATATGATAAAA  AAGATTATGACTGTGATTACGACAAAAAATATGATGACTAT  GATAAAAAATATTATGATCACGATAAAAAAGACTATGATTA  TGTTGTAGAGTATAAAAAGCATAAAAAACACTAC |
| *SOD* | ATGCCGACACACACGCTACCTGATTTGCCTTATGATCATGAA  GCATTGGCGCCGCACATAGACGCAAGAACGATGGAAATCCA  TCACGGAAAGCATCATCAAGGCTACGTGAATAACTTAAATGC  GGCGCTTGAAGGTCATCCAGAGCTTCAAGCAAAATCAGTTG  AAGAACTCGTCGCTGGCATTGATGCTGTTCCCGAGGCCATCA  GAACCGCGGTACGCAACAACGGCGGAGGGCATGCCAATCAT  TCCCTGTTTTGGCTTTCAATGTCTCCTCAGGGCGGGGGAGCG  CCAGACGGAGCTTTAGCCACTGCACTTGCAAATACATTTGGG  AGTTTCGGCGATTTTAAAGAACAGCTGACAGCCGCAAGCCT  CGCCCGATTCGGATCGGGATGGGGCTGGCTGGTCGTCACTTC  AGGCGGTGAGCTGGGTGTGTACAGCACAGCTAATCAAGACA  ATCCGTATATGCAGGGAGATGTGCCGATTTTAGGTGTAGACGT  ATGGGAGCACGCCTATTATTTAAACTATCAAAACCGCCGGCCG  GATTACTTGGCTGCTTGGTGGTCTGTTGTTGATTGGGACGAAG  TGGCAAGGCGTTTTGATGCGACGAGATAA |

**Figure S1.** Assessment of the vitro cytotoxic effects of Ethanol (EtOH) and SEVs on Aml-12 cells using the cck-8 assay. (A) Cytotoxicity of Aml-12 cells following exposure to varying concentrations of EtOH for a 4-hour period during the establishment of the in vitro model. (B) Cytotoxicity of Aml-12 cells upon 24-hour co-incubation with different concentrations of SEVs. (C) Cytotoxicity of Aml-12 cells determined after initial treatment with 100 mM EtOH for 4 hours to establish the cell model, followed by a subsequent 24-hour co-incubation with SEVs.

**Figure S2.** In vivo biodistribution of DiR-labeled SEVs in mice 1, 6, 12, 24 h after oral gavage.

**Figure S3.** Zeta potential and particle size distribution of SEVs under different storage temperatures. (A) Zeta potential distribution of SEVs in the initial state (SEVs-80°C), after 24-hour incubation at 4°C (SEVs-4°C), and after 24-hour incubation at 37°C (SEVs-37°C). (B-C) Particle size distribution curves of SEVs after 24-hour incubation at 4°C (B) and 37°C (C).

**Figure S4.** SOD enzymatic activity and structural integrity of CotC-SOD in SEVs before and after simulated gastrointestinal digestion. (A) Quantitative analysis of SOD activity in SEVs (pre-digestion), SGD-SEVs (post-simulated gastrointestinal digestion), and free SOD. Data are presented as mean±SEM. (B) Western blot analysis of His-tag (targeting 31 kDa CotC-SOD fusion protein) in SEVs and SGD-SEVs samples.

Figure S5. Uncropped Western blot images and ImageJ-based quantification of Nrf2, GAPDH and HO-1

Figure S6. Alterations in enzymatic activity and protein integrity of free SOD after simulated gastrointestinal digestion

(A) Quantitative comparison of SOD activity between native free SOD (SOD) and simulated gastrointestinal-digested free SOD (S-SOD), with extremely significant intergroup difference (****, p < 0.0001). (B) SDS-PAGE profiling to verify protein degradation of native free SOD (SOD) and digested free SOD (S-SOD).
